# P-1368. High prevalence of class 1 integrons in carbapenem resistant *Enterobacterales* in Taiwan

**DOI:** 10.1093/ofid/ofae631.1545

**Published:** 2025-01-29

**Authors:** Susan Shin-Jung Lee, Hui-Ling Hsia, Yi-ting Lee, Hsi-Hsun Lin

**Affiliations:** Kaohsiung Veterans General Hospital, Kaohsiung, Kaohsiung, Taiwan (Republic of China); Kaohsiung Veterans General Hospital, Kaohsiung, Kaohsiung, Taiwan (Republic of China); Kaohsiung Veterans General Hospital, Kaohsiung, Kaohsiung, Taiwan (Republic of China); Kaohsiung Veterans General Hospital, Kaohsiung, Kaohsiung, Taiwan (Republic of China)

## Abstract

**Background:**

The prevalence of integron in carbapenem resistant *Enterobacterales* is not well studied. This study aimed to investigate the prevalence and resistance gene cassettes of class 1 integron in carbapenem resistant *Enterobacterales*.

**Fig. 1.**
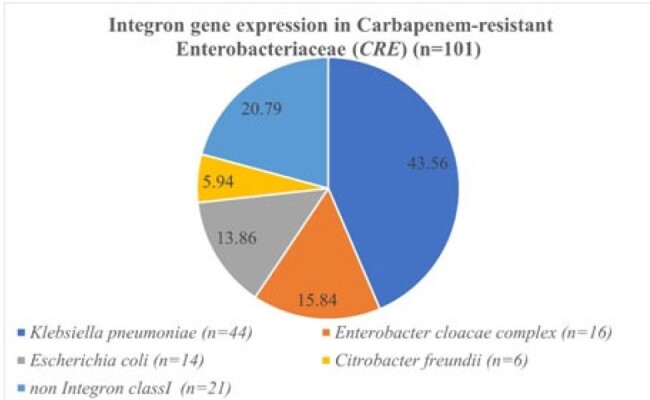
Integron gene expression in Carbapenem-resistant Enterobacterales (CRE)(N=101).

**Methods:**

In total, 101 carbapenem resistant *Enterobacterales* strains were collected

through 2020 to2023. Genomic DNA was extractedand PCR method was performed for detection of class 1 integron gene cassettes. Sequences were performed by an automatic sequencer with Applied Biosystems 3730xl and alignment in GenBank.

**Figure 2.**
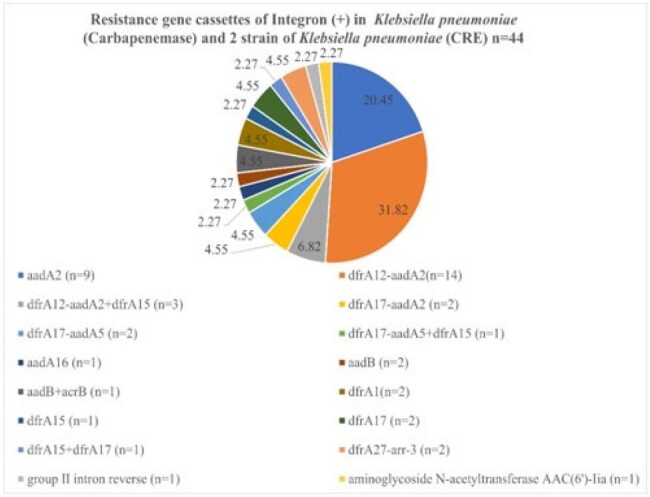
Resistance gene cassettes of Integron(+) in carbapemase-producing and non-carbapenemase producing carbapenem-resistant Klebsiella pneumoniae

**Results:**

The prevalence of class 1 integron cassette of all Carbapenem resistant *Enterobacterales* (CRE) was 79.21% (80/101), and was high as 83% (44/53) in carbapenem resistant *Klebsiella pneumoniae*.The most common resistant gene cassettes wer edfrA12-aadA2 (20 %), following by dfrA17-aadA5 (17.5%) and aadA2 (11.25%). No carbapenemase gene was detected in class 1 integron gene cassettes. However, acriflavine resistance B (AcrB) gene, which is a multiple efflux transporter, wasdetected in 4 strains.

**Table 1.**
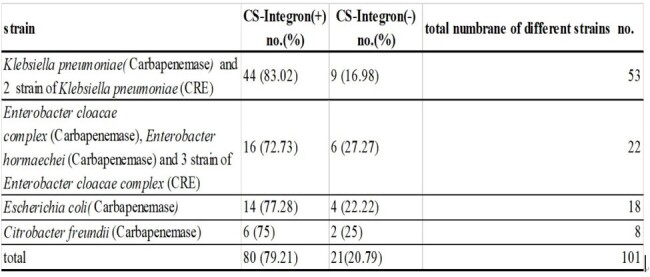
The expression patterns of integron genes (+) and integron genes (-) cassettes in different Carbapenem-resistant Enterobacterales (CRE).

**Conclusion:**

Our study revealed high prevalence of type I integron gene cassettes in carbapenemase resistance *Enterobacterales*. The role of AcrB gene need to be further studied.

**Figure:**
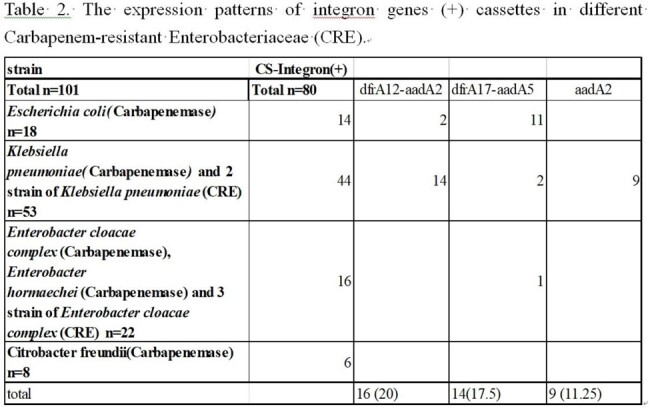

**Disclosures:**

**All Authors**: No reported disclosures

